# Metabolic alteration of urinary steroids in pre- and post-menopausal women, and men with papillary thyroid carcinoma

**DOI:** 10.1186/1471-2407-11-342

**Published:** 2011-08-08

**Authors:** Man Ho Choi, Ju-Yeon Moon, Sung-Hee Cho, Bong Chul Chung, Eun Jig Lee

**Affiliations:** 1 Future Convergence Research Division, Korea Institute of Science and Technology, Seoul 136-791, Korea; 2 Department of Internal Medicine, Yonsei University College of Medicine, Seoul 120-752, Korea

**Keywords:** Steroids, Thyroid cancer, Menopause, Gender difference, GC-MS

## Abstract

**Background:**

To evaluate the metabolic changes in urinary steroids in pre- and post-menopausal women and men with papillary thyroid carcinoma (PTC).

**Methods:**

Quantitative steroid profiling combined with gas chromatography-mass spectrometry was used to measure the urinary concentrations of 84 steroids in both pre- (n = 21, age: 36.95 ± 7.19 yr) and post-menopausal female (n = 19, age: 52.79 ± 7.66 yr), and male (n = 16, age: 41.88 ± 8.48 yr) patients with PTC. After comparing the quantitative data of the patients with their corresponding controls (pre-menopause women: n = 24, age: 33.21 ± 10.48 yr, post-menopause women: n = 16, age: 49.67 ± 8.94 yr, male: n = 20, age: 42.75 ± 4.22 yr), the levels of steroids in the patients were normalized to the mean concentration of the controls to exclude gender and menopausal variations.

**Results:**

Many urinary steroids were up-regulated in all PTC patients compared to the controls. Among them, the levels of three active androgens, androstenedione, androstenediol and 16α-hydroxy DHEA, were significantly higher in the pre-menopausal women and men with PTC. The corticoid levels were increased slightly in the PTC men, while progestins were not altered in the post-menopausal PTC women. Estrogens were up-regulated in all PTC patients but 2-hydroxyestrone and 2-hydroxy-17β-estradiol were remarkably changed in both pre-menopausal women and men with PTC. For both menopausal and gender differences, the 2-hydroxylation, 4-hydroxylation, 2-methoxylation, and 4-methoxylation of estrogens and 16α-hydroxylation of DHEA were differentiated between pre- and post-menopausal PTC women (*P *< 0.001). In particular, the metabolic ratio of 2-hydroxyestrone to 2-hydroxy-17β-estradiol, which could reveal the enzyme activity of 17β-hydroxysteroid dehydrogenase, showed gender differences in PTC patients (*P *< 1 × 10^-7^).

**Conclusions:**

These results are expected be helpful for better understanding the pathogenic differences in PTC according to gender and menopausal conditions.

## Background

Papillary thyroid carcinoma (PTC) is the commonest of all thyroid carcinomas and is well-differentiated. The incidence of PTC is three times higher in women than men [1,2] but this gender difference decreases after menopause [3]. The gender differences and menopausal conditions in the incidence of PTC suggest that the pathogenesis and development of PTC might be affected by sex steroids, particularly androgens and estrogens [3-11].

The biological activity of estrogens is differentiated by an interaction with both estrogen receptors α and β (ERα and ERβ). Especially, the level of ERα is higher in PTC cells than other thyroid carcinoma cells and normal cells [12]. In addition, the level of ERα is higher in pre-menopausal women than in post-menopausal women and men with PTC [7]. The androgen receptor (AR), which mediates the biological activity of androgens, can be identified in human thyroid cells [13-16]. The levels of AR in thyroid cells are higher in men than in women, and higher in PTC cells than in normal cells [14-16].

Although sex steroids including estrogens and androgens might be associated with both the growth and progression of PTC, the changes in steroids along with gender differences and menopause in PTC have not been well defined. No data is available on the metabolic alteration of steroid profiling between pre- and post-menopausal women, and men with PTC. Therefore, in this study, 84 steroids (including 25 androgens, 17 estrogens, 23 corticoids, 14 progestins, and 5 sterols) in urine samples obtained from pre- and post-menopausal women, and men with PTC were evaluated against the corresponding control groups matched for gender and menopausal condition using gas chromatography-mass spectrometry (GC-MS)-based steroid signatures [17]. Firstly, the urinary levels of steroids in the PTC patients and their controls in the three groups (pre- and post-menopausal women and men) were measured and significance in the individual groups was evaluated using a Student's *t*-test. Secondly, the steroid levels in each patient group were normalized to those of the corresponding controls to exclude the nature of menopausal and gender differences. The change in normalized steroids profiling between the three groups was evaluated by one-way variance analysis (one-way ANOVA). Finally, the metabolic patterns of steroids in the three groups were interpreted by partial least-squares-discrimination analysis (PLS-DA), and examined by ANOVA.

## Methods

### Subjects and sample-collection

Urine samples were collected from pre-menopausal women (n = 21, age: 36.95 ± 7.19 yr, BMI: 23.72 ± 4.69 kg/m^2^), post-menopausal women (n = 19, age: 52.79 ± 7.66 yr, BMI: 24.46 ± 2.76 kg/m^2^) and men (n = 16, age: 41.88 ± 8.48 yr, BMI: 25.52 ± 3.31 kg/m^2^) with PTC as well as from healthy subjects as the controls for the pre-menopausal women (n = 24, age: 33.21 ± 10.48 yr, BMI: 23.52 ± 3.94 kg/m^2^), post-menopausal women (n = 16, age: 49.67 ± 8.94 yr, BMI: 23.02 ± 3.38 kg/m^2^) and men (n = 20, age: 42.75 ± 4.22 yr, BMI: 24.58 ± 2.49 kg/m^2^) at the Severance Hospital (Seoul, Korea). All study subjects underwent the same diagnostic procedures, *i.e*., ultrasound and fine needle aspiration (FNA) as detailed by the American Joint Committee on Cancer staging. Patients with a history of cancer of the cervix, breast, endometrium, or head and neck, and respiratory papillomatosis as well as the post-menopausal women, who have received the estrogen replacement therapy, were excluded. The PTC not invaded the adjacent tissues or showed no spread to nearby lymph nodes. The healthy gender- and age-matched controls had no evidences of benign or malignant thyroid diseases. The first morning urine samples were collected after fasting for at least 12 hours and the patient samples were taken before thyroidectomy. All subjects showed normal thyroid function (T3: 104.11 ± 19.1 ng/dL, T4: 11.16 ± 2.56 μg/dL, TSH: 1.02 ± 0.31 μIU/mL) and were not treated with or exposed to any drugs including the contraceptive. The experimental protocol (4-2009-0424) was approved by the IRB Committee of the Human Research Protection Center at the Severance Hospital and an informed consent was signed by all subjects. The levels of urinary steroids were calibrated by the creatinine values using Jaffé's method [18].

### Chemicals and materials

The reference standards of the 84 steroids examined in this study (Table 1) were obtained from Sigma (St. Louis, MO), Steraloids (Newport, RI), and NARL (Pumble, Australia). The internal standards, 16,16,17-*d*_3_-testosterone and methyltestosterone for 25 androgens, 2,4,16,16-*d*_4_-estradiol for 17 estrogens, 9,11,12,12-*d*_4_-cortisol for 23 corticoids, 2,2,4,6,6,17α,21,21,21-*d*_9_-progesterone and 2,2,4,6,6,21,21,21-*d*_8_-17α-hydroxyprogesterone for 14 progestins, and 2,2,3,4,4,6-*d*_6_-cholesterol for 5 sterols were purchased from NARL and C/D/N isotopes (Pointe-Claire, Quebec, Canada). For solid-phase extraction (SPE), an Oasis HLB cartridge (3 mL, 60 mg; Waters, Milford, MA) was preconditioned with 3 mL of methanol followed by 3 mL of deionized water. Sodium acetate (reagent grade), acetic acid (glacial, 99.99+%) and L-ascorbic acid (reagent grade) were obtained from Sigma. A solution of β-glucuronidase was purchased from Roche Diagnostics GmbH (Mannheim, Germany). The trimethylsilylating (TMS) agents, *N*-methyl-*N*-trifluorotrimethylsilyl acetamide (MSTFA), ammonium iodide (NH_4_I), and dithioerythritol (DTE) were purchased from Sigma. All organic solvents used were of analytical or HPLC grade and were purchased from Burdick & Jackson (Muskegan, MI). Deionized water was prepared using a Milli-Q purification system (Millipore; Billerica, MA).

**Table 1:** Concentrations of urinary steroids in pre-menopausal PTC women and control

Steroids	Concentration^a ^(mean ± SD)		*P*-value
	
	Controls (n = 24)	PTC (n = 21)	Controls : PTC
***Androgens***			
Dihydrotestosterone	65.79 ± 47.43	118.67 ± 61.89	< 0.005
Epidihydrotestosterone	20.21 ± 16.70	44.85 ± 18.85	< 0.0007
Dehydroepiandrosterone	151.34 ± 84.40	216.29 ± 93.00	< 0.02
Testosterone	64.73 ± 45.54	116.60 ± 24.77	< 0.03
Epitestosterone	82.61 ± 49.75	109.66 ± 42.13	NS^b^
5α-androstan-3α,17β-diol	137.28 ± 90.96	202.60 ± 259.73	NS
5α-androstan-3β,17β-diol	20.00 ± 11.75 (3)	42.54 (1)	NC^c^
5β-androstan-3α,17β-diol	204.52 ± 149.53	248.07 ± 336.60	NS
5β-androstan-3α,17α-diol	70.50 ± 49.49	107.26 ± 105.68	NS
5β-androstan-3β,17α-diol	5.42 ± 1.69 (4)	6.30 ± 1.45 (3)	NC
5α-androstan-3β,17α-diol	21.17 ± 14.55	41.20 ± 28.43	NS
5α-androstan-3α,17α-diol	33.64 ± 26.33	53.18 ± 41.56	NS
5β-androstan-3β,17β-diol	8.75 (1)	36.84 ± 53.75 (4)	NC
Androstenedione	59.17 ± 50.56	96.93 ± 39.56	< 0.01
Androstenediol	51.71 ± 31.61	96.27 ± 33.98	< 0.0002
Androsterone	7826.70 ± 5459.40	24675.61 ± 23074.97	< 0.004
Etiocholanolone	7074.80 ± 4371.84	21993.33 ± 14558.44	< 0.0002
11-keto-androsterone + 11-keto-etiocholanolone	439.95 ± 320.79	2048.15 ± 1749.63	< 0.0005
11β-hydroxyandrosterone	2413.61 ± 1411.53	5197.95 ± 4501.89	< 0.01
11β-hydroxyetiocholanolone	1185.01 ± 873.09	2598.36 ± 1740.91	< 0.003
5β-dihydrotestosterone	3.97 ± 2.92	21.71 ± 9.26	< 0.00008
16α-hydroxy-DHEA	127.76 ± 67.50	285.48 ± 147.21	< 0.0002
Epiandrosterone	33.26 ± 26.59	49.45 ± 29.48	NS
5α-Androstanedione	36.43 ± 15.15 (11)	59.43 ± 12.34 (4)	NC
***Estrogens***			
Estrone	71.16 ± 60.17	165.12 ± 138.61	< 0.008
17β-estradiol	20.78 ± 19.43	52.64 ± 42.82	< 0.008
Estriol	69.12 ± 55.60	197.03 ± 403.37	NS
2-hydroxyestrone	298.38 ± 272.41	126.11 ± 63.97	< 0.007
2-hydroxy-17β-estradiol	84.88 ± 60.34	283.06 ± 247.77	< 0.002
4-hydroxyestrone	72.78 ± 57.37	119.25 ± 54.27	< 0.02
4-hydroxy-17β-estradiol	37.69 ± 30.34	91.53 ± 49.54	< 0.0003
2-methoxyestrone	53.79 ± 96.35	94.21 ± 48.47	NS
2-methoxy-17β-estradiol	28.85 ± 23.85	43.09 ± 18.79	NS
4-methoxyestrone	36.30 ± 32.13	61.86 ± 29.64	< 0.04
4-methoxy-17β-estradiol	29.38 ± 19.95	61.79 ± 37.91	< 0.02
17-epiestriol	18.07 ± 8.07	97.53 ± 56.54	< 0.003
16-epiestriol	37.43 ± 16.97	83.48 ± 68.51	< 0.02
17α-estradiol	2.18 ± 1.98 (2)	ND	NC
2-hydroxyestriol	2.37 ± 2.74	85.99 ± 63.04	< 0.002
16-keto-17β-estradiol + 16α-hydroxyestrone	20.79 ± 11.75	70.92 ± 81.33	< 0.01
***Corticoids***			
Cortisol	276.27 ± 348.99	194.98 ± 121.70	NS
Allodihydrocortisol	38.87 ± 26.40	24.27 ± 18.96	NS
Corticosterone	37.33 ± 28.36	54.26 ± 24.03	NS
Allodihydrocorticosterone	33.18 ± 26.19	42.48 ± 18.15	NS
Dihydrodeoxycorticosterone	97.57 ± 145.03	90.54 ± 53.46	NS
11-deoxycorticosterone	43.68 ± 27.30	48.25 ± 19.29	NS
11-deoxycortisol	59.55 ± 42.52	53.88 ± 37.00	NS
Cortisone	700.66 ± 691.13	828.94 ± 783.61	NS
Allotetrahydrocortisol	6987.19 ± 6000.57	14415.42 ± 17559.79	NS
21-deoxycortisol	69.17 ± 45.07	89.61 ± 80.03	NS
11-dehydrocorticosterone	131.08 ± 121.05	92.97 ± 93.87	NS
Tetrahydrodeoxycortisol	502.93 ± 336.31	547.57 ± 293.54	NS
Tetrahydrocortisone	12623.53 ± 9093.38	14081.85 ± 11546.70	NS
Tetrahydrocortisol	6038.33 ± 4364.60	10488.74 ± 9218.05	NS
Tetrahydrodeoxycorticosterone	72.07 ± 90.74	76.89 ± 88.78	NS
Tetrahydrocorticosterone	353.41 ± 307.84	589.34 ± 321.77	< 0.02
11-dehydrotetrahydrocorticosterone	1403.89 ± 610.28	992.50 ± 677.32	NS
α-cortolone	4989.28 ± 3076.48	5749.11 ± 3617.93	NS
β-cortolone	2030.16 ± 1204.44	2745.91 ± 1714.17	NS
20α-dihydrocortisone	340.09 ± 272.98	423.71 ± 252.98	NS
α-cortol	921.30 ± 645.85	884.54 ± 634.69	NS
β-cortol	2296.04 ± 1382.42	2602.54 ± 1638.69	NS
20α-dihydrocortisol	209.95 ± 260.36	130.72 ± 105.39	NS
***Progestins***			
Pregnenolone	44.36 ± 33.00	73.78 ± 32.67	< 0.008
Progesterone	45.35 (1)	194.29 (1)	NC
5β-dihydroprogesterone	7.23 ± 5.09	7.88 (1)	NC
5α-dihydroprogesterone	53.83 ± 26.13	20.36 ± 25.60	< 0.04
20α-hydroprogesterone	125.50 ± 189.43	153.23 ± 126.74	NS
Epipregnanolone	19.10 ± 17.42	29.50 ± 12.29	NS
Pregnanolone	717.51 ± 900.30	1305.74 ± 2701.21	NS
Allopregnanolone	198.24 ± 255.64	188.66 ± 240.25	NS
Isopregnanolone	33.41 ± 13.44	73.87 ± 50.59	< 0.02
Pregnanediol	7025.27 ± 8837.04	14031.18 ± 31235.57	NS
Pregnanetriol	2874.08 ± 1477.34	5021.37 ± 4085.30	< 0.04
17α-hydroxypregnenolone	57.89 ± 42.96	87.99 ± 40.07	< 0.03
17α-hydroxyprogesterone	53.61 ± 43.13 (6)	97.95 ± 63.76 (2)	NC
11β-hydroxyprogesterone	938.19 ± 726.72	1557.51 ± 1150.75	< 0.05
***Sterols***			
Cholesterol	4601.20 ± 9878.96	7633.19 ± 8383.76	NS
Desmosterol	167.78 ± 64.99	327.76 ± 127.00	NS
Lanosterol	76.86 ± 82.14	78.06 (1)	NC
20α-hydroxycholesterol	59.65 ± 52.14	93.08 ± 24.37	NS
24S-hydroxycholesterol	53.36 ± 35.45	66.87 ± 30.67	NS

The number in the parentheses is the number of subject detected corresponding steroid.

^a^Concentrations are expressed as ng/g of creatinine (mean ± SD).

^b^NS, not significant.

^c^NC, not comparable.

### Sample preparation

Quantitative steroid profiling was performed using a previous technique [17]. Briefly, a urine sample (2 mL) spiked with 20 μL of 7 internal standards (*d*_3_-testosterone and *d*_4_-estradiol: 1 μg/mL, *d*_4_-cortisol and *d*_8_-17α-hydroxyprogesterone: 5 μg/mL, methyltestosterone, *d*_9_-progesterone and *d*_6_-cholesterol: 10 μg/mL) was loaded into the Oasis HLB™ SPE cartridge and washed with 2 mL water. After elution twice with 2 mL of methanol, the combined methanol was evaporated under a stream of nitrogen and 1 mL of 0.2 M acetate buffer (pH 5.2), 100 μL of 0.2% L-ascorbic acid, and 50 μL of β-glucuronidase were then added. After incubation at 55°C for 3 h, the solution was extracted twice with 2.5 mL of ethyl acetate: *n*-hexane (2:3, v/v). The combined organic solvents were evaporated using a N_2 _evaporator at 40°C and dried further in a vacuum desiccator over P_2_O_5_-KOH for at least 30 min. Finally, the dried residue was derivatized with MSTFA/NH_4_I/DTE (40 μL; 500:4:2, v/w/w) at 60°C for 20 min, and 2 μL of the resulting mixture was subjected to GC-MS in selected-ion monitoring (SIM) mode.

### Instrumental conditions

GC-MS was performed using an Agilent 6890 Plus gas chromatograph interfaced with a single-quadrupole Agilent 5975 MSD at an electron energy of 70 eV and ion source temperature of 230°C. Each sample (2 μL) was injected in split mode (10:1) at 280°C and separated through an Ultra-1 capillary column (25 m × 0.2 mm i.d., 0.33 μm film thickness; Agilent Technologies; Palo Alto, CA). The GC oven temperature was initially set at 215°C, then ramped to 260°C at 1°C/min, and finally increased to 320°C at 15°C/min and held for 1 min. The carrier gas was helium at a column head pressure of 210.3 kPa (column flow: 1.0 mL/min at oven temperature of 215°C). For quantitative analysis, the characteristic ions of each steroid were determined as their TMS derivatives. Peak identifications were achieved by comparing the retention times and matching the height ratios of the characteristic ions [17].

### Statistical analysis

The levels of urinary steroids are reported as the mean ± SD. The significance of these variables obtained from the controls and PTC groups was examined using a Student's *t*-test. The variables in PTC were normalized to the mean of the corresponding controls, and significance of the normalized three groups was also evaluated by one-way variance analysis (one-way ANOVA). A *P*-value < 0.01 was considered significant. Partial least squares-discrimination analysis (PLS-DA) with multivariate data analysis software SIMCA-P (version 11.0, Umetrics Inc., Sweden) was used for clustering the study groups.

### Multivariate data analysis

The processed data was stored in either Excel spreadsheets or Comma Separated Values (CSV) formatted files and then imported into SIMCA software. The urinary steroid levels were classified by PLS-DA for pre- and post-menopausal female and male PTC patients after normalization. PLS-DA maximizes the covariance between the predicting data set, which is X of the numerical value of the targeted steroids, and Y of the class assignment.

The fraction of the variation in the Y variables "explained" by the selected components (R^2^Y), along with the fraction of the variation of Y's that can be "predicted" by a component according to cross validation (Q^2^Y) were calculated to plot and validate the model. After calculating the components for the PLS-DA scatter plot, the significant components were selected according to the rule embedded in SIMCA-P software, whereby Q^2 ^should be larger than zero for more than 100 observations and 0.05 for ≤ 100 observations. The PLS-DA plots were then displayed as the superposition of the highest two latent variables (t[1]/t[2] or p[1]/p[2] as X-, Y- axes), such that most of the association with dummy Y variables could be explained by the variations in X. In addition, the high coefficient values of R^2^Y and Q^2^Y revealed good discrimination. In this study, the urinary steroids were scaled and centered prior to PLS-DA. One point in the scatter plot represents the rates of steroid secretion. The metabolic patterns can be interpreted using visual images or the R^2^Y and Q^2^Y values.

## Results

### Urinary levels of steroids in PTC patients

The urinary levels of 84 steroids in the pre- and post-menopausal women and men with PTC, along with their corresponding controls were profiled quantitatively. Tables 1, 2 and 3 list the concentrations in the three PTC groups and their individual controls. In pre-menopausal PTC women, all estrogens including estrone (*P *< 0.008), 17β-estradiol (*P *< 0.008), 2-hydroxy-17β-estradiol (*P *< 0.002), 4-hydroxy-17β-estradiol (*P *< 0.0003), 17-epiestriol (*P *< 0.003), 2-hydroxyestriol (*P *< 0.002) were increased, whereas 2-hydroxyestrone was significantly decreased (*P *< 0.007). All androgens were also up-regulated in the PTC patients including the most active androgens, dihydrotestosterone (*P *< 0.005), androstenediol (*P *< 0.0002), and 16α-hydroxy DHEA (*P *< 0.0002). Progestins tended to be higher in the PTC patients, but the 5α-dihydroprogesterone levels were slightly lower (*P *< 0.04). Both corticoids and sterols were not significant (Table 1), whereas lanosterol was detectable in only one patient.

**Table 2:** Concentrations of urinary steroids in post-menopausal PTC women and control

Steroids	Concentration^a ^(mean ± SD)		*P*-value
	
	Controls (n = 16)	PTC (n = 19)	Controls : PTC
***Androgens***			
Dihydrotestosterone	71.99 ± 45.10	91.87 ± 57.97	NS^b^
Epidihydrotestosterone	38.16 ± 27.60	32.35 ± 58.50	NS
Dehydroepiandrosterone	165.46 ± 91.64	194.37 ± 109.70	NS
Testosterone	102.07 ± 64.62	196.74 ± 232.37	NS
Epitestosterone	92.02 ± 69.39	91.08 ± 60.77	NS^b^
5α-androstan-3α,17β-diol	87.09 ± 64.60	112.99 ± 94.87	NS
5α-androstan-3β,17β-diol	4.26 (1)	ND^c^	NC^d^
5β-androstan-3α,17β-diol	99.98 ± 74.50	109.73 ± 90.40	NS
5β-androstan-3α,17α-diol	67.53 ± 62.11	61.33 ± 68.37	NS
5β-androstan-3β,17α-diol	14.33 ± 13.01 (2)	9.58 ± 7.20 (4)	NC
5α-androstan-3β,17α-diol	22.27 ± 20.57 (2)	34.51 ± 28.74 (4)	NC
5α-androstan-3α,17α-diol	43.24 ± 52.23	37.50 ± 24.00	NS
5β-androstan-3β,17β-diol	ND	18.10 (1)	NC
Androstenedione	110.21 ± 88.05	112.78 ± 59.83	NS
Androstenediol	54.27 ± 26.28	61.30 ± 13.63	NS
Androsterone	4464.11 ± 1786.06	14974.73 ± 17229.72	< 0.02
Etiocholanolone	4850.22 ± 2075.09	13920.59 ± 15821.56	< 0.03
11-keto-androsterone + 11-keto-etiocholanolone	580.33 ± 297.69	1727.55 ± 2045.94	< 0.03
11β-hydroxyandrosterone	4499.84 ± 2455.83	5488.85 ± 4203.96	NS
11β-hydroxyetiocholanolone	1462.44 ± 1071.41	2629.38 ± 2439.84	NS
5β-dihydrotestosterone	4.79 ± 1.61	21.03 ± 9.54	< 0.00003
16α-hydroxy-DHEA	228.48 ± 172.71	271.17 ± 127.46	NS
Epiandrosterone	60.19 ± 61.75	70.25 ± 35.51	NS
5α-Androstanedione	72.20 ± 83.17 (4)	60.13 ± 24.16 (13)	NC
***Estrogens***			
Estrone	163.00 ± 176.41 (6)	63.72 ± 47.10 (17)	NC
17β-estradiol	14.24 ± 14.24 (4)	21.84 ± 19.84 (7)	NC
Estriol	56.96 ± 65.77 (6)	60.11 ± 57.72 (17)	NC
2-hydroxyestrone	306.99 ± 99.92 (5)	50.92 ± 30.20 (18)	NC
2-hydroxy-17β-estradiol	131.39 ± 166.91 (6)	168.07 ± 372.40 (17)	NC
4-hydroxyestrone	76.21 ± 15.24 (4)	42.27 ± 49.44 (15)	NC
4-hydroxy-17β-estradiol	33.90 ± 13.10 (4)	44.94 ± 23.08 (16)	NC
2-methoxyestrone	69.67 ± 77.40 (6)	101.47 ± 68.85 (7)	NC
2-methoxy-17β-estradiol	24.53 ± 11.18 (3)	60.09 ± 29.12 (7)	NC
4-methoxyestrone	32.32 (1)	85.60 ± 41.64 (15)	NC
4-methoxy-17β-estradiol	66.69 ± 75.09 (4)	55.39 ± 11.16 (4)	NC
17-epiestriol	254.59 (1)	94.42 ± 44.07 (8)	NC
16-epiestriol	74.15 ± 85.83 (6)	119.59 ± 63.08 (6)	NC
17α-estradiol	ND	ND	NC
2-hydroxyestriol	ND	90.89 ± 102.52 (5)	NC
16-keto-17β-estradiol + 16α-hydroxyestrone	28.01 ± 33.72 (6)	46.10 ± 24.21 (18)	NC
***Corticoids***			
Cortisol	235.02 ± 205.57	561.28 ± 345.19	NS
Allodihydrocortisol	71.42 ± 64.96	61.67 ± 84.03	NS
Corticosterone	20.74 ± 5.63	100.83 ± 85.62	< 0.02
Allodihydrocorticosterone	53.16 ± 46.48	66.19 ± 38.41	NS
Dihydrodeoxycorticosterone	20.41 ± 15.52	162.62 ± 155.03	< 0.02
11-deoxycorticosterone	101.66 ± 97.56	61.76 ± 29.06	NS
11-deoxycortisol	101.54 ± 102.04	45.92 ± 22.60	NS
Cortisone	980.80 ± 750.48	2205.39 ± 2667.76	NS
Allotetrahydrocortisol	9613.87 ± 6293.12	17105.92 ± 16369.40	NS
21-deoxycortisol	86.96 ± 32.86	97.31 ± 54.06	NS
11-dehydrocorticosterone	124.14 ± 75.34	194.61 ± 298.04	NS
Tetrahydrodeoxycortisol	794.79 ± 305.00	818.38 ± 581.02	NS
Tetrahydrocortisone	17811.52 ± 12271.25	16114.72 ± 12222.84	NS
Tetrahydrocortisol	8640.16 ± 4558.83	16040.33 ± 12750.63	< 0.05
Tetrahydrodeoxycorticosterone	56.16 ± 73.09	43.58 ± 61.64	NS
Tetrahydrocorticosterone	389.66 ± 207.67	780.31 ± 575.27	< 0.02
11-dehydrotetrahydrocorticosterone	877.74 ± 538.13	1445.62 ± 1602.49	NS
α-cortolone	4888.98 ± 2535.86	29149.71 ± 97516.67	NS
β-cortolone	2589.95 ± 927.40	5524.05 ± 11016.02	NS
20α-dihydrocortisone	375.30 ± 192.08	1766.88 ± 5192.38	NS
α-cortol	944.46 ± 362.03	5294.32 ± 17534.68	NS
β-cortol	2299.34 ± 908.51	8550.42 ± 23540.39	NS
20α-dihydrocortisol	172.51 ± 127.47	786.04 ± 2141.06	NS
***Progestins***			
Pregnenolone	66.45 ± 68.65	108.91 ± 65.30	NS
Progesterone	ND	47.86 (1)	NC
5β-dihydroprogesterone	13.35 ± 9.78	ND	NC
5α-dihydroprogesterone	38.87 ± 28.09 (2)	24.96 ± 37.35	NC
20α-hydroprogesterone	121.40 ± 105.37 (3)	276.33 ± 450.77	NC
Epipregnanolone	63.07 ± 63.10	37.64 ± 22.54	NS
Pregnanolone	652.71 ± 1018.03	489.54 ± 1157.06	NS
Allopregnanolone	147.26 ± 129.01	109.29 ± 187.61	NS
Isopregnanolone	60.44 ± 5.17 (3)	73.12 ± 43.22	NC
Pregnanediol	6959.54 ± 12541.46	3635.06 ± 9400.59	NS
Pregnanetriol	1559.68 ± 1314.30	2612.22 ± 3133.33	NS
17α-hydroxypregnenolone	85.37 ± 85.75	106.24 ± 57.67	NS
17α-hydroxyprogesterone	ND	133.63 ± 84.38	NC
11β-hydroxyprogesterone	1204.77 ± 516.99	6580.30 ± 17763.59	NS
***Sterols***			
Cholesterol	5103.58 ± 3075.92 (6)	5002.90 ± 3033.29	NC
Desmosterol	197.65 ± 67.86 (2)	ND	NC
Lanosterol	102.03 ± 125.18 (4)	32.83 ± 20.51 (3)	NC
20α-hydroxycholesterol	ND	86.56 ± 4.77 (2)	NC
24S-hydroxycholesterol	116.58 ± 101.37 (4)	54.63 ± 26.17 (7)	NC

The number in the parentheses is the number of subject detected corresponding steroid.

^a^Concentrations are expressed as ng/g of creatinine (mean ± SD).

^b^NS, not significant.

^c^ND, not detected.

^d^NC, not comparable.

**Table 3:** Concentrations of urinary steroids in the PTC men and control

Steroids	Concentration^a ^(mean ± SD)		*P*-value
	
	Controls (n = 20)	PTC (n = 16)	Controls : PTC
***Androgens***			
Dihydrotestosterone	91.39 ± 62.43	132.14 ± 92.41	NS^b^
Epidihydrotestosterone	20.74 ± 16.54	28.45 ± 14.21	NS
Dehydroepiandrosterone	144.93 ± 58.60	180.74 ± 85.79	NS
Testosterone	101.16 ± 89.00	271.30 ± 270.50	NS
Epitestosterone	181.57 ± 93.89	177.67 ± 111.44	NS
5α-androstan-3α,17β-diol	381.95 ± 183.28	320.57 ± 198.42	NS
5α-androstan-3β,17β-diol	3.70 ± 3.66	83.37 ± 99.92	NS
5β-androstan-3α,17β-diol	432.61 ± 362.77	607.93 ± 1056.85	NS
5β-androstan-3α,17α-diol	55.09 ± 46.59	109.91 ± 153.06	NS
5β-androstan-3β,17α-diol	ND^c^	4.92 (1)	NC^d^
5α-androstan-3β,17α-diol	31.72 ± 39.33	54.92 ± 80.93	NS
5α-androstan-3α,17α-diol	31.13 ± 28.59	40.00 ± 63.40	NS
5β-androstan-3β,17β-diol	2.51 (1)	ND	NC
Androstenedione	40.66 ± 21.23	92.10 ± 51.74	< 0.002
Androstenediol	28.68 ± 12.20	82.31 ± 38.09	< 0.00005
Androsterone	10087.69 ± 4603.39	26110.67 ± 11663.35	< 0.00006
Etiocholanolone	8256.47 ± 3997.97	24745.49 ± 16868.98	< 0.002
11-keto-androsterone + 11-keto-etiocholanolone	531.90 ± 598.85	1660.94 ± 1191.92	< 0.003
11β-hydroxyandrosterone	4009.02 ± 2754.69	7037.26 ± 4372.55	< 0.02
11β-hydroxyetiocholanolone	1295.60 ± 1325.27	2087.71 ± 1610.32	NS
5β-dihydrotestosterone	3.01 ± 1.85	17.67 ± 20.67	< 0.02
16α-hydroxy-DHEA	79.37 ± 43.01	186.33 ± 63.29	< 0.000005
Epiandrosterone	24.21 ± 10.91	46.90 ± 33.66	< 0.02
5α-Androstanedione	11.29 (1)	24.39 (1)	NC
***Estrogens***			
Estrone	39.69 ± 12.89	60.18 ± 29.20	< 0.02
17β-estradiol	12.91 ± 12.56	15.05 ± 10.68	NS
Estriol	24.62 ± 11.84	44.79 ± 31.46	< 0.03
2-hydroxyestrone	50.27 ± 25.50	91.83 ± 55.12	< 0.02
2-hydroxy-17β-estradiol	195.56 ± 113.59	64.98 ± 38.05	< 0.00007
4-hydroxyestrone	51.38 ± 26.59	59.70 ± 24.78	NS
4-hydroxy-17β-estradiol	18.29 ± 7.63	42.05 ± 13.14	< 0.00009
2-methoxyestrone	22.78 ± 13.07	42.46 ± 20.94	< 0.006
2-methoxy-17β-estradiol	19.41 ± 9.13	27.37 ± 14.25	NS
4-methoxyestrone	17.78 ± 7.24	34.29 ± 16.50	< 0.003
4-methoxy-17β-estradiol	19.00 ± 9.90	28.78 ± 11.78	< 0.03
17-epiestriol	42.81 (1)	49.14 ± 5.76 (2)	NC
16-epiestriol	28.28 ± 19.49	34.70 ± 16.18	NS
17α-estradiol	0.92 (1)	ND	NC
2-hydroxyestriol	0.57 ± 0.77	40.82 ± 69.05	NS
16-keto-17β-estradiol + 16α-hydroxyestrone	9.99 ± 4.63	22.93 ± 10.98	< 0.0003
***Corticoids***			
Cortisol	183.91 ± 160.81	487.21 ± 482.28	< 0.03
Allodihydrocortisol	38.21 ± 28.01	17.59 ± 8.64	< 0.005
Corticosterone	33.47 ± 32.89	56.57 ± 91.42	NS
Allodihydrocorticosterone	22.53 ± 10.10	29.87 ± 13.54	NS
Dihydrodeoxycorticosterone	32.30 ± 37.87	134.38 ± 105.38	< 0.02
11-deoxycorticosterone	28.74 ± 15.27	28.78 ± 13.55	NS
11-deoxycortisol	37.14 ± 16.35	32.66 ± 15.58	NS
Cortisone	511.43 ± 377.83	1580.13 ± 1320.58	< 0.006
Allotetrahydrocortisol	9510.49 ± 4735.51	20666.67 ± 12334.24	< 0.003
21-deoxycortisol	63.27 ± 29.68	88.63 ± 45.03	NS
11-dehydrocorticosterone	110.59 ± 105.33	148.87 ± 147.64	NS
Tetrahydrodeoxycortisol	405.48 ± 214.37	756.82 ± 539.85	< 0.03
Tetrahydrocortisone	11055.45 ± 5289.49	18809.78 ± 11894.93	< 0.03
Tetrahydrocortisol	6091.23 ± 2812.78	16371.31 ± 10629.87	< 0.002
Tetrahydrodeoxycorticosterone	24.76 ± 15.31	37.50 ± 24.52	NS
Tetrahydrocorticosterone	433.03 ± 604.84	659.73 ± 380.95	NS
11-dehydrotetrahydrocorticosterone	993.99 ± 1013.47	1127.02 ± 618.49	NS
α-cortolone	4030.85 ± 1778.81	6992.04 ± 3748.34	< 0.009
β-cortolone	2284.12 ± 1041.75	3306.24 ± 1662.55	< 0.05
20α-dihydrocortisone	252.58 ± 107.63	431.88 ± 202.66	< 0.005
α-cortol	838.32 ± 319.86	1180.62 ± 550.93	< 0.04
β-cortol	2181.33 ± 886.33	3316.76 ± 1830.36	< 0.04
20α-dihydrocortisol	87.90 ± 57.63	188.16 ± 129.88	< 0.01
***Progestins***			
Pregnenolone	27.08 ± 13.26	41.82 ± 15.30	**< 0.02**
Progesterone	22.47 ± 11.45 (2)	47.85 ± 10.86 (3)	NC
5β-dihydroprogesterone	7.90 ± 7.77	11.18	NS
5α-dihydroprogesterone	24.37 ± 11.29	28.45 ± 35.05	NS
20α-hydroprogesterone	35.96 ± 24.53	76.35 ± 54.00	< 0.01
Epipregnanolone	13.87 ± 8.23	16.85 ± 6.83	NS
Pregnanolone	251.57 ± 121.38	559.35 ± 835.31	NS
Allopregnanolone	55.53 ± 25.97	92.13 ± 129.62	NS
Isopregnanolone	32.57 ± 11.39	47.07 ± 24.81	NS
Pregnanediol	1896.17 ± 1077.27	5669.51 ± 10408.32	NS
Pregnanetriol	2704.00 ± 1146.97	3975.05 ± 1957.07	< 0.04
17α-hydroxypregnenolone	39.84 ± 22.13	58.70 ± 38.95	NS
17α-hydroxyprogesterone	15.19 (1)	39.82 ± 19.99	NC
11β-hydroxyprogesterone	818.94 ± 373.38	2518.86 ± 1739.51	< 0.002
***Sterols***			
Cholesterol	2028.74 ± 1781.46	3851.26 ± 2743.30	< 0.04
Desmosterol	216.36 ± 88.55	421.52 ± 622.80	NS
Lanosterol	58.32 ± 73.89	43.89 ± 30.30	NS
20α-hydroxycholesterol	49.98 (1)	85.15 (1)	NC
24S-hydroxycholesterol	111.65 ± 207.00	27.96 ± 15.91	NS

The number in the parentheses is the number of subject detected corresponding steroid.

^a^Concentrations are expressed as ng/g of creatinine (mean ± SD).

^b^NS, not significant.

^c^ND, not detected.

^d^NC, not comparable.

In contrast, the levels of corticosterone (*P *< 0.02), dihydrodeoxycorticosterone (*P *< 0.02), tetrahydrocortisol (*P *< 0.05) and tetrahydrocorticosterone (*P *< 0.02) were slightly higher in the post-menopausal PTC women than in the corresponding controls (Table 2). Most estrogens could be up-regulated in the groups studied but their urinary levels were undetectable in many post-menopausal women in both the patient and control groups. The levels of 5β-dihydrotestosterone were significantly higher in the patient group (*P *< 0.00003), but this was not clinically meaningful. The other steroids, progestins and sterols were not significant.

In PTC men, the 4-hydroxy-17β-estradiol (*P *< 0.00009), 2-methoxyestrone (*P *< 0.006) and 4-methoxyestrone (*P *< 0.003) levels were significantly higher in the patients, whereas 2-hydroxy-17β-estradiol levels were lower (*P *< 0.00007; Table 3). In particular, increased levels of 2-hydroxyestrone and decreased levels of 2-hydroxy-17β-estradiol in the patients were reversible with the results obtained from the PTC women. Although the corticoids levels in the pre- and post-menopausal women groups were not associated with PTC, the levels of most corticoids were higher the patients except for allodihydrocortisol (38.21 ± 28.01 ng/g creatinine for controls; 17.59 ± 8.64 for patients, *P *< 0.005). Some active androgens including androstenedione (*P *< 0.002), androstenediol (*P *< 0.00005) and 16α-hydroxy-DHEA (*P *< 0.000005) in the patients were associated with PTC. The levels of progestins and sterols were not remarkable.

In all cases, androgens were up-regulated in the patient groups compared to the control groups. Among the urinary androgens studied, five androgens (androsterone, etiocholanolone, 11-keto-androsterone, 11-keto-etiocholanolone and 5β-DHT), which are abundant steroids in humans, were significantly higher in all patient groups (Tables 1, 2, and 3). Active androgens including androstenedione, androstenediol and 16α-hydroxy DHEA were significantly higher in the pre-menopausal women and men with PTC, but not in the post-menopausal PTC women. Estrogens were also up-regulated in all patients compared to the controls. In particular, the 2-hydroxyestrone level was lower in the pre-menopausal PTC women (*P *< 0.007) but higher in the PTC men (*P *< 0.02). In contrast, 2-hydroxy-17β-estradiol was higher in the pre-menopausal PTC women (*P *< 0.002), whereas its level was lower in PTC men (*P *< 0.00007). Two hydroxylated estrogens at C-2 showed different biological actions between genders. Corticoids were slightly higher in the PTC men and post-menopausal PTC women but not in pre-menopausal PTC women. Progestins were also higher in the patients except for post-menopausal PTC women. In this study, no sterols were shown to be significant in the PTC groups.

### Group differences in the steroid metabolism

To exclude the nature of menopause and gender differences between the patient groups studied, all steroid levels in the PTC groups were normalized to the mean values of the corresponding controls, and the different pathogenesis of PTC was then compared according to the menopausal and gender conditions. Statistical analysis was performed using PLS-DA with normalized levels of steroids in the three groups (Additional file. 1). Using the visual inspecting metabolic patterns of steroids and the coincidences between R^2^Y and Q^2^Y, the pre- and post-menopausal women, and male groups with PTC were clustered individually (Additional file 1A). Their metabolic patterns were discriminated clearly (R^2^Y = 0.727, Q^2^Y = 0.558). The loading plot of PLS-DA, which is complement to the score plot due to the transposed matrix calculation, was also performed to identify the possible urinary biomarkers. Each data point represents one particular steroid with the relationship between the different PTC patients (Additional file 1B). The metabolic differentiation between the groups complemented the results listed in Additional file 2.

Several possible biomarkers for individual PTC groups were selected (Additional file 2). The levels of androstenediol (*P *< 0.005) was significantly higher in the PTC men than in both PTC women groups, whereas 16α-hydroxy DHEA was higher in both pre-menopausal and men with PTC (*P *< 0.002). The normalized androgens levels in PTC men were significantly higher than normalized pre- or post-menopausal PTC women (androstenediol, *P *< 0.005; 16α-hydroxy-DHEA, *P *< 0.002) (Additional file 2). In both PTC women groups, corticosterone (*P *< 0.005) and dihydrodeoxycorticosterone (*P *< 0.009) were higher in the post-menopausal PTC women group than in the pre-menopausal PTC women and PTC men, whereas the post-menopausal PTC women showed lower levels of 11-deoxycorticosterone (*P *< 0.003). The normalized corticoids levels (corticosterone, *P *< 0.005; dihydrodeoxycorticosterone, *P *< 0.009; 11-deoxycorticosterone, *P *< 0.003) of post-menopausal PTC women were significantly higher than in the other groups (Additional file 2). In progestins, only epipregnanolone showed a significance in post-menopausal PTC women compared to both pre-menopausal and men with PTC (*P *< 0.0004). In the case of 24S-hydroxycholesterol, its urinary concentrations were not remarkable in any of the groups compared, but the normalized values were significantly higher in the pre-menopausal PTC women (*P *< 0.000002). Estrogens (estrone, *P *< 0.0001, 2-hydroxy-17β-estradiol, *P *< 0.002; 4-hydroxyestrone, *P *< 0.001; 4-hydroxy-17β-estradiol, *P *< 0.006; 17-epiestriol, *P *< 0.0008) in the pre-menopausal PTC women were significantly higher than those of post-menopausal women and men with PTC, whereas 2-hydroxyestrone was significantly higher in PTC men (*P *< 0.0000001).

**Figure 1:**
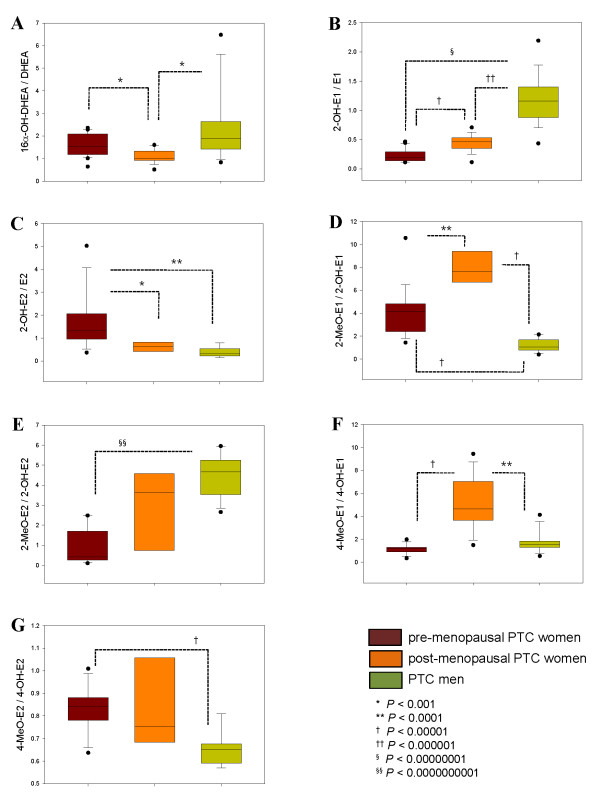
**Enzyme activities based on the normalized levels of urinary steroids from pre- and post-menopausal women and men with PTC**. The level differences significant at the *P *< 0.01 were selected. *Line within the box *represents the median, *lower boundary of the box *indicates 25%, and the *upper boundary of the box *indicates 75%. *Whiskers above and below *indicate the maximum and minimum steroid levels. *Dots above and below *indicate the plot outliers with the 10^th ^and 90^th ^percentiles.

**Figure 2:**
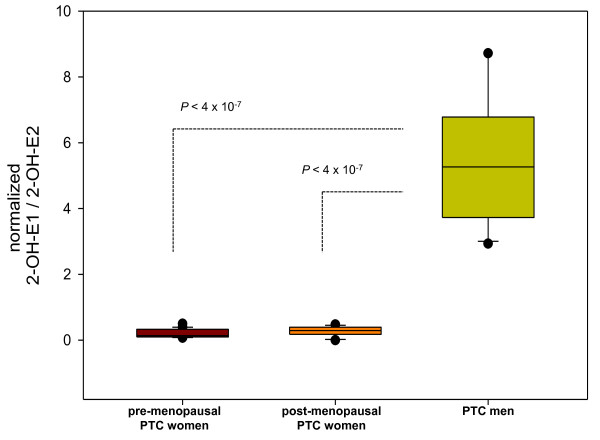
**Altered 2-hydroxyestrone to 2-hydroxy-17β-estradiol metabolic ratio in sex difference**. Its metabolic ratio could reveal 17β-hydroxysteroid dehydrogenase in the estrogen metabolism. *Line within the box *represents the median, *lower boundary of the box *indicates 25%, and the *upper boundary of the box *indicates 75%. *Whiskers above and below *indicate the maximum and minimum steroid levels. *Dots above and below *indicate the plot outliers with the 10^th ^and 90^th ^percentiles.

To demonstrate the enzyme activities in the steroid metabolism, the ratio of the steroid metabolite to precursor was examined. Box plots of the altered steroids normalized in all PTC patients showed differences in these ratios (Figure 1). The 16α-hydroxylation of DHEA was differentiated between pre-menopausal women and men with PTC (Figure 1A). In the case of 2-hydroxylation, all PTC groups were significantly discriminated with the 2-hydroxyestrone to estrone ratio (Figure 1B), whereas the 2-hydroxy-17β-estradiol to 17β-estradiol ratio could differentiate the pre-menopausal PTC women with post-menopausal PTC women and PTC men (Figure 1C). For 2-methoxylation, all groups were differentiated with the ratio of 2-methoxyestrone to 2-hydroxyestrone (Figure 1D), whereas 17β-estradiol represented the pre-menopausal women and men with PTC groups (Figure 1E). 4-methoxylation with the 4-methoxyestrone to 4-hydroxyestrone ratio showed differences between post-menopausal PTC women and the other two PTC groups (Figure 1F), whereas 4-hydroxy-17β-estradiol differentiated between pre-menopausal women and men with PTC (Figure 1G). There were statistically significant differences found in the estrogen metabolites ratio of 2-hydroxyestrone to 2-hydroxy-17β-estradiol, which could indicate 17β-hydroxysteroid dehydrogenase (17β-HSD) between women and men with PTC (< 4 × 10^-7^; Figure 2).

## Discussion

Although an understanding of the pathogenesis and development in PTC has been investigated, there is no data available on the metabolic alteration of steroids according to gender and menopausal condition. The metabolic profiling of urinary steroids in pre- and post-menopausal women, and men with PTC were achieved. Due to gender and menopausal variations in steroid biosynthesis, each patient group was normalized to the corresponding control group before comparing the patient groups.

ERα causes cell proliferation and progress in PTC [7,12,19]. The level of ERα is higher in PTC cells than normal cells, and is higher in pre-menopausal women than in both post-menopausal women and men with PTC [7]. The urinary levels of estrogens in pre-menopausal women were investigated first. The concentrations of all estrogens in the pre-menopausal PTC women were higher than the controls, except for 2-hydroxyestrone, which was significantly lower (Table 1). The extent to which the 2-hydroxyestrogens are active or can form active or genotoxic metabolites is controversial [20,21]. 2-hydroxyestrone has been characterized as the "good estrogen" [22]. In addition, the normalized levels of estrone, 2-hydroxy-17β-estradiol, 4-hydroxyestrone, and 4-hydroxy-17β-estradiol were also significantly higher in pre-menopausal PTC women than normalized post-menopausal women and men with PTC, whereas the level of 2-hydroxyestrone was significantly up-regulated in PTC men (Additional file 2). In addition, the estrogen metabolism has been shown to potentiate the growth of hormone related cancers, such as breast and cervical cancer [23,24]. 2-Hydroxyestrone and 16α-hydroxyestrone as the oxidative metabolites of estrone have different biological actions with anti-proliferation and proliferative effects, respectively, in hormone related cancers [24,25].

In this study, 16α-hydroxyestrone was not exactly quantified because of its co-elution with 16-keto-17β-estradiol during chromatographic separation. In addition, 4-hydroxyestrone generates reactive oxygen species (ROS) as a source of oxidative stress through estrogen metabolic redox cycling [26] and increases the formation of endogenous carcinogens [24]. Here, 4-hydroxyestrogens was increased significantly, whereas 2-hydroxyestrone was decreased in both PTC women groups (Tables 1, 2 and 3). In general, 2-hydroxyestrone does not have estrogenic activity peripherally and may be anti-estrogenic, leading to an anti-proliferative effect on estrogen-sensitive cells as the good estrogen [27].

AR causes cell proliferation in PTC [15] and its activity is higher in men than women, and is more active in PTC cells than normal cells [14-16]. Nevertheless, the concentrations of most estrogens, except for 2-hydroxy-17β-estradiol, and androgens (androstenedione, androstenediol, androsterone, etiocholanolone, and 16α-hydroxy-DHEA) in PTC men were higher than the controls (Table 3). The normalized levels of androstenediol and 16α-hydroxy-DHEA in PTC men were significantly higher than normalized pre- or post-menopausal PTC women through the higher androgen levels in men (Additional file 2). In addition, the levels of AR were increased by a testosterone treatment in PTC [28]. In this study, the normalized levels of testosterone in PTC men were more significant than pre- or post-menopausal PTC women through AR activation, and the normalized levels of androstenedione and androstenediol as a precursor of testosterone in PTC males were more significant than in the other PTC groups (Additional file 2).

Based on their metabolic actions, corticoids are divided by glucocorticoids and mineralocorticoids, [29] and they are generally considered to simulate lipogenesis, and accelerate fatty acid synthesis [30,31]. Therefore, the concentrations of both corticoids series, such as corticosterone, dihydrodeoxycorticosterone, tetrahydrocortisol, and tetrahydrocorticosterone in post-menopausal PTC women are higher than its controls (Table 2). However, the normalized levels of mineralocorticoids including corticosterone, dihydrodeoxycorticosterone, and 11-deoxycorticosterone in post-menopausal PTC women were significantly higher than in the other groups (Additional file 2).

Many steroids and metabolic ratios indicate both menopausal and gender differences in PTC progression (Figure 1) but their levels are discriminated by the degree of up-regulation in PTC patients. In particular, 2-hydroxyestrone and 2-hydroxy-17β-estradiol show reversible actions between pre-menopausal women and men with PTC (Tables 1 and 3). 2-hydroxyestrone stimulates the anti-proliferation of cancer cells directly, whereas the effect of 2-hydroxy-17β-estradiol on carcinomas is more complex. In addition, 2-hydroxy-17β-estradiol was up-regulated in the pre-menopausal PTC women and its production may result in oxidative DNA damage and apoptosis in human mammalian cells [32]. Inter-conversion between 2-hydroxyestrone and 2-hydroxy-17β-estradiol catalyzed by 17β -HSD was significantly altered in PTC men against both pre- and post-menopausal women with PTC (Figure 2). The expression of 17β-HSD is not only specific in thyroid disorders [7], but may indicate the gender differences. However, its reversible actions in men have not been investigated.

## Conclusions

Although both most androgens and estrogens were increased in the pre-menopausal women with PTC in this study, hydroxylation and methoxylation with estrogens explained more the metabolic changes between all groups studied. In this cross-sectional study, the thyroid cancer risk might be associated with lower 2-hydroxyation activities of either estrone or 17β-estradiol in women and men. Overall, the 2-hydroxyestrone to 2-hydroxy-17β-estradiol metabolic ratio, which indicates the activity of 17β-HSD, may in fact be gender differences in the thyroid cancer progression. These results may help better understand the pathogenesis of PTC according to gender and menopausal conditions.

## Competing interests

The authors declare that they have no competing interests.

## Authors' contributions

MHC carried out the preparing manuscript and the experimental design. JYM developed the analytical assay and performed the statistical analysis. SHC participated in the design of the study and carried out the steroid profiling. BCC conceived of the study, and participated in its design and coordination and helped to draft the manuscript. EJL participated in preparing the experimental protocol, clinical diagnosis and sampling. All authors revised the manuscript and approved the final version.

## Pre-publication history

The pre-publication history for this paper can be accessed here:

http://www.biomedcentral.com/1471-2407/11/342/prepub

## Supplementary Material

Additional file 1 **PLS-DA score (A) and loading (B) plots from a profiling of 84 urinary steroids**. The steroid profiling of pre- and post-menopausal female, and male patients with papillary thyroid carcinoma (PTC) was conducted. The levels of urinary steroids were normalized to the mean values of individual control groups and then differentiated by one-way ANOVA. The abbreviation of the steroids in the loading plot was obtained from reference 20. Click here for file

Additional file 2 **Levels of normalized urinary steroids in the pre- and post-menopausal women and men with PTC**. To exclude the nature of menopause and gender differences between the patient groups studied, all steroid levels in the PTC groups were normalized to the mean values of the corresponding controls, and the normalized differentiation proposed several possible biomarkers. Click here for file
