# Supplementary material for: Metabolic alteration of urinary steroids in pre- and post-menopausal women, and men with papillary thyroid carcinoma
Source: BMC Cancer. 2011 Aug 8;11:342. doi: 10.1186/1471-2407-11-342 (PMC3199870; doi:10.1186/1471-2407-11-342)
Supplement: Additional file 2 — Levels of normalized urinary steroids in the pre- and post-menopausal women and men with PTC. To exclude the nature of menopause and gender differences between the patient groups studied, all steroid levels in the PTC groups were normalized to the mean values of the corresponding controls, and the normalized differentiation proposed several possible biomarkers. [file 1471-2407-11-342-S2.PDF]

Steroids	Normalized level[a] (mean ± SD)			*P*-value
	Pre-menopausal female	Post-menopausal female	Male	
Androgens				
Dihydrotestosterone	1.80 ± 0.94	1.28 ± 0.81	1.45 ± 1.01	NS[b]
Epidihydrotestosterone	2.22 ± 0.93	1.53 ± 0.85	1.37 ± 0.69	NS
Dehydroepiandrosterone	1.43 ± 0.61	1.17 ± 0.66	1.25 ± 0.59	NS
Testosterone	1.80 ± 0.38	1.93 ± 2.28	2.68 ± 2.67	< 0.04
Epitestosterone	1.33 ± 0.50	0.99 ± 0.66	0.98 ± 0.61	NS
5α-androstan-3α,17β-diol	1.48 ± 1.89	1.30 ± 1.09	0.84 ± 0.52	NS
5α-androstan-3β,17β-diol	2.13 (1)	-	22.55 ± 27.03	NC[C]
5β-androstan-3α,17β-diol	1.21 ± 1.65	1.10 ± 0.90	1.41 ± 2.44	NS
5β-androstan-3α,17α-diol	1.52 ± 1.50	0.91 ± 1.01	2.00 ± 2.78	NS
5β-androstan-3β,17α-diol	1.16 ± 0.27 (3)	0.83 ± 0.63	-	NC
5α-androstan-3β,17α-diol	1.95 ± 1.34	1.16 ± 0.97	1.73 ± 2.55	NS
5α-androstan-3α,17α-diol	1.58 ± 1.24	0.87 ± 0.55	1.29 ± 2.04	NS
5β-androstan-3β,17β-diol	4.21 ± 6.14 (4)	-	-	NC
Androstenedione	1.64 ± 0.67	1.02 ± 0.54	2.27 ± 1.27	< 0.03
Androstenediol	1.76 ± 0.77	1.13 ± 0.25	2.87 ± 1.33	< 0.005
Androsterone	3.15 ± 2.95	3.35 ± 3.86	2.59 ± 1.16	NS
Etiocholanolone	3.11 ± 2.06	2.87 ± 3.26	3.00 ± 2.04	NS
11-keto-androsterone + 11-keto-etiocholanolone	4.66 ± 3.98	2.98 ± 3.53	3.12 ± 2.24	NS
11β-hydroxyandrosterone	2.15 ± 1.87	1.22 ± 0.93	1.76 ± 1.09	NS
11β-hydroxyetiocholanolone	2.19 ± 1.47	1.80 ± 1.67	1.61 ± 1.24	NS
5β-dihydrotestosterone	5.47 ± 2.33	4.39 ± 1.99	5.88 ± 6.87	NS
16α-hydroxy-DHEA	2.23 ± 1.15	1.19 ± 0.56	2.35 ± 0.80	< 0.002
Epiandrosterone	1.49 ± 0.89	1.17 ± 0.59	1.94 ± 1.39	< 0.02
5α-Androstanedione	1.63 ± 0.34 (4)	0.83 ± 0.33	2.16 (1)	NC
Estrogens				
Estrone	2.32 ± 1.95	0.39 ± 0.29	1.52 ± 0.74	< 0.0001
17β-estradiol	2.53 ± 2.06	1.53 ± 1.39	1.17 ± 0.83	NS
Estriol	2.85 ± 5.84	1.06 ± 1.01	1.82 ± 1.28	NS
2-hydroxyestrone	0.42 ± 0.21	0.17 ± 0.10	1.83 ± 1.10	< 0.0000001
2-hydroxy-17β-estradiol	3.33 ± 2.92	1.28 ± 2.83	0.33 ± 0.19	< 0.002
4-hydroxyestrone	1.64 ± 0.75	0.55 ± 0.65	1.16 ± 0.48	< 0.001
4-hydroxy-17β-estradiol	2.43 ± 1.31	1.33 ± 0.68	2.30 ± 0.72	< 0.006
2-methoxyestrone	1.75 ± 0.90	1.46 ± 0.99	1.86 ± 0.92	NS
2-methoxy-17β-estradiol	1.49 ± 0.65	2.45 ± 1.19	1.41 ± 0.73	< 0.02

4-methoxyestrone	1.70 ± 0.82	2.65 ± 1.29	1.93 ± 0.93	< 0.04
4-methoxy-17β-estradiol	2.10 ± 1.29	0.83 ± 0.17	1.51 ± 0.62	NS
17-epiestriol	5.40 ± 3.13	0.37 ± 0.17	1.15 ± 0.13 (2)	< 0.0008
16-epiestriol	2.23 ± 1.83	1.61 ± 0.85	1.23 ± 0.57	NS
17α-estradiol	-	-	-	NC
2-hydroxyestriol	36.30 ± 26.61	-	71.49 ± 120.93	NC
16-keto-17β-estradiol + 16α-hydroxyestrone	3.41 ± 3.91	1.65 ± 0.86	2.30 ± 1.10	< 0.02
Corticoids				
Cortisol	0.75 ± 0.76	2.68 ± 2.76	2.65 ± 2.62	< 0.02
Allodihydrocortisol	0.62 ± 0.49	0.86 ± 1.18	0.46 ± 0.23	NS
Corticosterone	1.45 ± 0.64	4.86 ± 4.13	1.69 ± 2.73	< 0.005
Allodihydrocorticosterone	1.28 ± 0.55	1.24 ± 0.72	1.33 ± 0.60	NS
Dihydrodeoxycorticosterone	0.93 ± 0.55	7.97 ± 7.60	4.16 ± 3.26	< 0.009
11-deoxycorticosterone	1.10 ± 0.44	0.61 ± 0.29	1.00 ± 0.47	< 0.003
11-deoxycortisol	0.90 ± 0.62	0.45 ± 0.22	0.88 ± 0.42	NS
Cortisone	1.25 ± 1.11	2.38 ± 2.74	3.09 ± 2.58	< 0.05
Allotetrahydrocortisol	2.06 ± 2.51	1.88 ± 1.69	2.17 ± 1.30	NS
21-deoxycortisol	1.30 ± 1.16	1.12 ± 0.62	1.40 ± 0.71	NS
11-dehydrocorticosterone	0.71 ± 0.72	1.57 ± 2.40	1.35 ± 1.34	NS
Tetrahydrodeoxycortisol	1.09 ± 0.58	1.03 ± 0.73	1.87 ± 1.33	< 0.02
Tetrahydrocortisone	1.12 ± 0.91	0.90 ± 0.69	1.70 ± 1.08	< 0.04
Tetrahydrocortisol	1.74 ± 1.53	1.86 ± 1.48	2.69 ± 1.75	NS
Tetrahydrodeoxycorticosterone	1.07 ± 1.23	0.78 ± 1.10	1.51 ± 0.99	NS
Tetrahydrocorticosterone	1.67 ± 0.91	2.00 ± 1.48	1.52 ± 0.88	NS
11-dehydrotetrahydrocorticosterone	0.71 ± 0.48	1.65 ± 1.83	1.13 ± 0.62	NS
α-cortolone	1.15 ± 0.73	5.96 ± 19.95	1.73 ± 0.93	NS
β-cortolone	1.35 ± 0.84	2.13 ± 4.25	1.45 ± 0.73	NS
20α-dihydrocortisone	1.25 ± 0.74	4.71 ± 13.84	1.71 ± 0.80	NS
α-cortol	0.96 ± 0.69	5.61 ± 18.57	1.41 ± 0.66	NS
β-cortol	1.13 ± 0.71	3.72 ± 10.24	1.52 ± 0.84	NS
20α-dihydrocortisol	0.62 ± 0.50	4.56 ± 12.41	2.14 ± 1.48	NS
Progestins				
Pregnenolone	1.66 ± 0.74	1.64 ± 0.98	1.43 ± 0.69	NS
Progesterone	4.28 (1)	-	2.13 ± 0.48 (3)	NC
5β-dihydroprogesterone	1.09 (1)	-	1.42 (1)	NC
5α-dihydroprogesterone	0.38 ± 0.48	0.64 ± 0.96	1.17 ± 1.44	NS
20α-hydroprogesterone	1.22 ± 1.01	2.28 ± 3.71	2.12 ± 1.50	NS

Epipregnanolone	1.54 ± 0.64	0.60 ± 0.36	1.21 ± 0.49	< 0.0004
Pregnanolone	1.82 ± 3.76	0.75 ± 1.77	2.21 ± 3.32	NS
Allopregnanolone	0.95 ± 1.21	0.74 ± 1.27	1.66 ± 2.33	NS
Isopregnanolone	2.21 ± 1.51	1.21 ± 0.72	1.45 ± 0.76	NS
Pregnanediol	2.10 ± 4.54	0.67 ± 1.50	2.99 ± 5.49	NS
Pregnanetriol	1.84 ± 1.40	1.77 ± 2.02	1.47 ± 0.72	NS
17α-hydroxypregnenolone	1.52 ± 0.69	1.24 ± 0.68	1.47 ± 0.98	NS
17α-hydroxyprogesterone	1.83 ± 1.19 (2)	-	2.62 ± 1.32	NC
11β-hydroxyprogesterone	1.66 ± 1.23	5.46 ± 14.74	3.08 ± 2.12	NS
Sterols				
Cholesterol	1.66 ± 1.82	0.98 ± 0.59	1.90 ± 1.35	NS
Desmosterol	1.95 ± 0.76 (4)	-	1.95 ± 2.88 (5)	NC
Lanosterol	1.02 (1)	0.32 ± 0.20 (2)	0.75 ± 0.52 (4)	NS
20α-hydroxycholesterol	1.56 ± 0.41 (3)	-	1.70 (1)	NC
24S-hydroxycholesterol	1.25 ± 0.57	0.47 ± 0.22	0.25 ± 0.14	< 0.000002

The number in the parentheses is the number of subject detected corresponding steroid.

[a]Normalized levels are expressed as the ratio of individual patients concentration versus mean values of the corresponding controls (mean ± SD).

[b]NS, not significant.

[c]NC, not comparable.
