# Supplementary material for: Metabolic alteration of urinary steroids in pre- and post-menopausal women, and men with papillary thyroid carcinoma
Source: BMC Cancer. 2011 Aug 8;11:342. doi: 10.1186/1471-2407-11-342 (PMC3199870; doi:10.1186/1471-2407-11-342)
Supplement: Additional file 1 — PLS-DA score (A) and loading (B) plots from a profiling of 84 urinary steroids. The steroid profiling of pre- and post-menopausal female, and male patients with papillary thyroid carcinoma (PTC) was conducted. The levels of urinary steroids were normalized to the mean values of individual control groups and then differentiated by one-way ANOVA. The abbreviation of the steroids in the loading plot was obtained from reference 20. [file 1471-2407-11-342-S1.PDF]

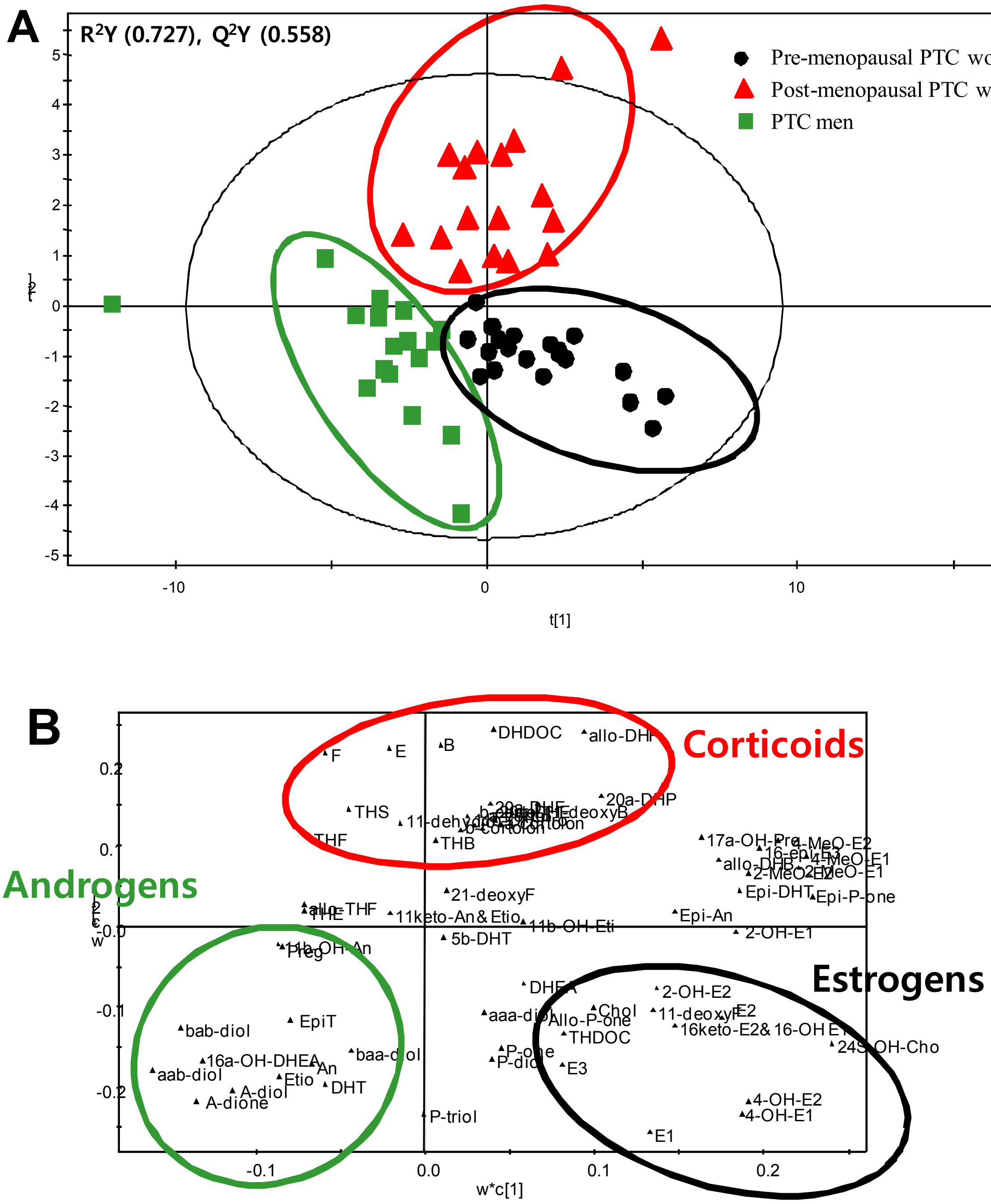
A
R2Y (0.727), Q2Y (0.558)
Pre-menopausal PTC women
Post-menopausal PTC women
PTC men
t[1]
-10
0
10
5
4
3
2
1
0
-1
-2
-3
-4
-5
B
Corticoids
Androgens
Estrogens
DHDOC
allo-DHF
F
E
B
THS
THF
THB
20a-DHP
21-deoxyF
11keto-An & Etio
11b-OH-Eti
5b-DHT
17a-OH-Preg
Epi-DHT
Epi-P-one
Epi-An
2-OH-E1
2-OH-E2
DHEA
aaa-diol
Allo-P-one
Chol
THDOC
11-deoxyF
16keto-E2& 16-OH E1
24S-OH-Cho
P-one
P-diol
E3
4-OH-E2
4-OH-E1
E1
P-triol
EpiT
bab-diol
16a-OH-DHEA
An
baa-diol
aab-diol
Etio
A-diol
DHT
A-dione
-0.1
0.0
0.1
0.2
w*c[1]
